# Fermentative hydrogen production using pretreated microalgal biomass as feedstock

**DOI:** 10.1186/s12934-018-0871-5

**Published:** 2018-02-14

**Authors:** Jianlong Wang, Yanan Yin

**Affiliations:** 1 Collaborative Innovation Center for Advanced Nuclear Energy Technology, INET, Energy Science Building, Tsinghua University, Beijing, 100084 People’s Republic of China; 2 Beijing Key Laboratory of Radioactive Waste Treatment, Tsinghua University, Beijing, 100084 People’s Republic of China

**Keywords:** Microalgae, Biohydrogen, Pretreatment, Fermentation

## Abstract

Microalgae are simple chlorophyll containing organisms, they have high photosynthetic efficiency and can synthesize and accumulate large quantities of carbohydrate biomass. They can be cultivated in fresh water, seawater and wastewater. They have been used as feedstock for producing biodiesel, bioethanol and biogas. The production of these biofuels can be integrated with CO_2_ mitigation, wastewater treatment, and the production of high-value chemicals. Biohydrogen from microalgae is renewable. Microalgae have several advantages compared to terrestrial plants, such as higher growth rate with superior CO_2_ fixation capacity; they do not need arable land to grow; they do not contain lignin. In this review, the biology of microalgae and the chemical composition of microalgae were briefly introduced, the advantages and disadvantages of hydrogen production from microalgae were discussed, and the pretreatment of microalgal biomass and the fermentative hydrogen production from microalgal biomass pretreated by different methods (including physical, chemical, biological and combined methods) were summarized and evaluated. For the production of biohydrogen from microalgae, the economic feasibility remains the most important aspect to consider. Several technological and economic issues must be addressed to achieve success on a commercial scale.

## Background

The fossil fuels are depleting and resulting in serious environment issues. Hydrogen gas is regarded as a potential candidate for a future energy economy. Hydrogen is the only carbon-free fuel, with water as its final combustion product. Therefore the application of hydrogen will greatly contribute to the reduction of the energy-related environmental issues, such as greenhouse emission or acid rain [1, 2].

Biohydrogen is defined as hydrogen produced biologically, most commonly by algae, bacteria and archaea from both cultivation and from waste organic materials [3]. Most biologically produced hydrogen in the biosphere is evolved in microbial fermentation processes. These organisms decompose organic matter to carbon dioxide and hydrogen.

**Fig. 1:**
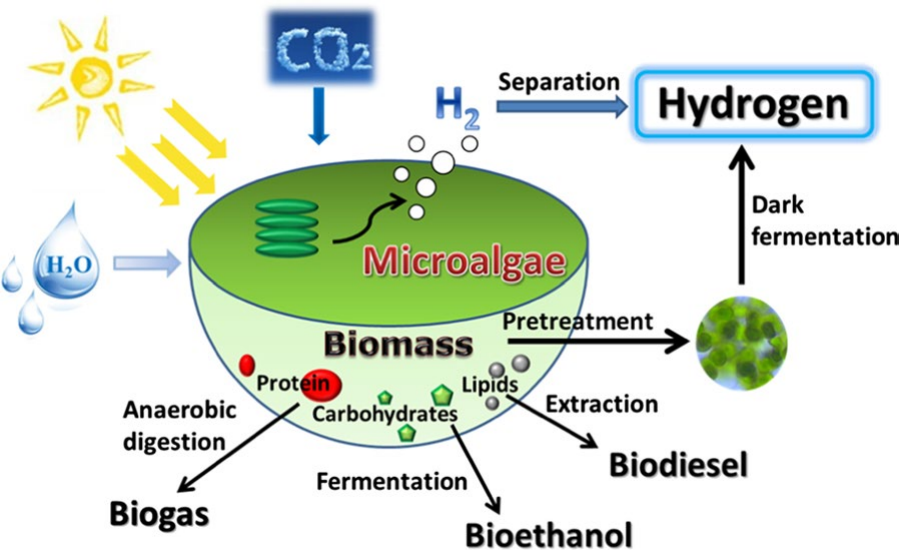
Potential pathways from microalgae to biofuels

Microalgal biomass, being rich in carbohydrates, has great potential as feedstock for the production of various biofuels such as biodiesel, bioethanol, biohydrogen and biogas (Fig. 1), in an economically effective and environmentally friendly way [4]. Microalgae are a high-potential source of biomass for the production of food, industrial materials, pharmaceuticals and energy [5].

Microalgae like cyanobacteria and green algae can produce biohydrogen after derivation of their photosynthetic metabolism. Besides, microalgae can also be used as feedstock for biohydrogen production by microbial dark fermentation.

Biohydrogen from microalgae is renewable. The production of biohydrogen by microalgae through photo-fermentation is of interest, because it generates hydrogen gas from the most plentiful resources, light and water. However, the adaptation of the algae to an anaerobic atmosphere is prerequisite. Unfortunately, hydrogen production by this process is quite ineffective since the simultaneously produced oxygen would inhibit the hydrogenase enzyme. Therefore, accumulation of oxygen will stop the hydrogen production process.

The production of biohydrogen from microalgae through dark fermentation has received increasing attention in recent years [6]. However, biohydrogen potentials are usually low and a pretreatment step is often required to convert polymeric carbohydrates into monomeric sugars, to increase the microbial accessibility and further the biohydrogen production. Thus, physical, chemical and biological pretreatments are usually employed in order to facilitate carbohydrates de-polymerization and enhance biohydrogen production from microalgae.

The present mini-review will briefly introduce the biohydrogen production from microalgal biomass through dark fermentation, focusing on the pretreatments of microalgae to enhance hydrogen production.

## Biology of microalgae

**Fig. 2:**
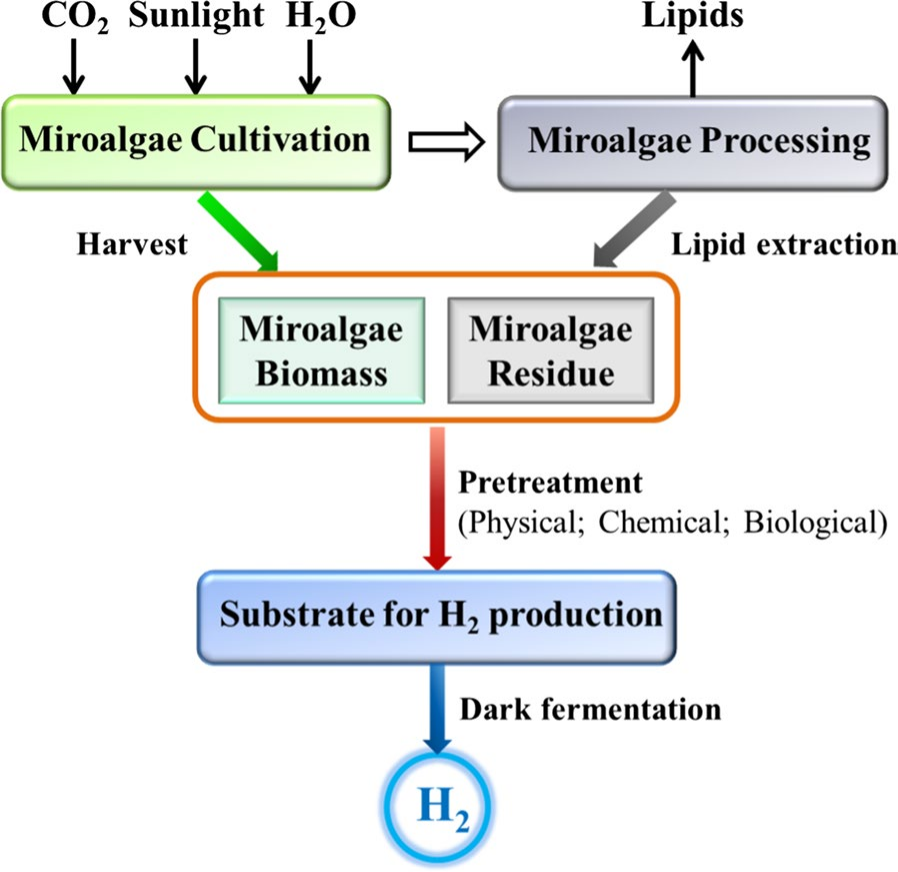
Roadmap from microalgae to hydrogen

Microalgae in this review refer to all microscopic oxygenic phototrophs. Microalgae are primitive plant, which are one of the oldest life forms on earth. They are lack of roots, stems and leaves, have chlorophyll a as their primary photosynthetic pigment. Microalgae are commonly photosynthetic organisms that primarily use water, carbon dioxide, and sunlight to produce biomass and oxygen (Fig. 2).

Microalgae are a diverse group of prokaryotic and eukaryotic photosynthetic microorganisms, which are normally found in marine and freshwater habitats. They can be grouped into prokaryotic microalgae (Cyanobacteria), eukaryotic microalgae (green algae Chlorophyta), red algae (Rhodophyta), and diatoms (Bacillariophyta), which are capable of growing rapidly due to their low nutrient requirement and simple structure. Besides to natural environments, microalgae can be cultivated in freshwater, seawater, and wastewater within open ponds (raceway) and closed photo-bioreactors.

Microalgae structures are primarily for energy conversion, and their simple development makes them to adapt to prevailing environmental conditions.

Microalgae are autotrophic, heterotrophic and mixotrophic. The autotrophic algae require only inorganic carbon source such as CO_2_, salts and a light energy source for growth; while the heterotrophic ones are non-photosynthetic, they require an external source of organic compounds as an energy source; the mixotrophic algae are capable of performing photosynthesis and acquiring exogenous organic nutrients. For autotrophic algae, photosynthesis is a key component of their survival, whereby they convert solar light and CO_2_ into adenosine triphosphate (ATP) and O_2_, which is then used in respiration to produce energy to support growth.

## Microalgal chemical composition

Components of microalgae vary according to their species and cultivation environment. Microalgae contain approximately 50% carbon by dry weight, which is typically derived from carbon dioxide. Production of 100 g of microalgal biomass can fix about 183 g of carbon dioxide.

In terms of chemical composition, microalgal biomass is mainly composed of proteins, carbohydrates and lipids. In general, proteins account for 40–60% of dry biomass, followed by carbohydrate (20–30%) and lipids (10–20%). Table 1 presents the general compositions of different microalgae [7–9].

**Table 1:** General composition of different microalgae (% of dry matter)

Microalgae	Protein	Carbohydrate	Lipid
*Anabaena cylindrica*	43–56	25–30	4–7
*Chlamydomonas reinhardtii*	48	17	21
*Chlorella vulgaris*	51–58	12–17	14–22
*Dunaliella salina*	57	32	6
*Porphyridium cruentum*	28–39	40–57	9–14
*Scenedesmus obliquus*	50–56	10–17	12–14
*Spirulina maxima*	60–71	13–16	6–7
*Synechococcus* sp.	63	15	11

Table 1 shows that the distribution of biochemical fractions of a microalgae cell is as follows: proteins 28–71%, carbohydrates 10–57%, lipids 4–22%. It is worth noting that the figures presented in Table 1 are estimates, since the proportion of individual cell constituents largely depends on environmental parameters. The chemical composition of microalgae is high variable, largely depending on species, environmental conditions and cultivation methods. For instance, nutritional limitation and deprivation can induce and maximize lipid and carbohydrates synthesis by changing the metabolic strategies of microalgae. Microalgal cells tend to synthesize lipid instead of the starch at nitrogen-limited and high light conditions. In addition to these three major components, microalgal cells also contain small amount of nucleic acids (1–5%), and other valuable components (foe example, pigments, anti-oxidants, fatty acids and vitamins) [10–14].

## Advantages and disadvantages of hydrogen production from microalgae

The components of microalgae are valuable for a wide range of applications. Carbohydrates in microalgae can exist in the form of glucose and some polysaccharides like starch, agar, carrageenan, etc., which are considered to be an appropriate feedstock for generation of various fermentation products. Algal lipids are composed of glycerol, sugars or bases esterified to saturated or unsaturated fatty acids, which can be used for biodiesel production. The related long-chain fatty acids, pigments, and proteins have their own nutraceutical and pharmaceutical applications.

Comparing with the cellulose-based biomass and waste activated sludge produced from wastewater treatment plant, microalgae are a relatively new energy source. They have many advantages, for example, they have high growth rate with the fixation of CO_2_, cultivation of microalgae can be beneficial to the environment by combining with wastewater treatment, they can be easily used as substrate with high carbohydrate content and simple structure and so on (Table 2) [7, 9, 10, 14].

**Table 2:** Advantages and disadvantages of microalgae as feedstock for biohydrogen production

Advantages	**Disadvantages**
1. *High growth rate*	1. *Low biomass concentration*
Microalgae can proliferate rapidly and are capable of all year round production, and be obtained in large amount easily, which makes it possible to satisfy the massive demand on biofuels using limited land resources without causing potential biomass deficit. Their exponential growth rates can double their biomass in periods as short as 3.5 h	The low biomass concentration in the microalgal culture, in combination with the small size of algal cells, makes the harvest of algal biomasses relatively costly
2. *Superior CO*_*2*_ *fixation capacity*	2. *Large water content*
Microalgae are quite efficient in utilizing inorganic carbon sources to synthesize cell biomass, and their tolerance to high CO_2_ content in gas streams allows high-efficiency CO_2_ mitigation (1 kg of dry algal biomass utilize about 1.83 kg of CO_2_)	The large water content of harvested algal biomass suggests that its drying process would be energy-consuming
3. *Benefit to the environment*	3. *Higher capital cost*
The cultivation of microalgae does not require herbicides or pesticides application. Nitrous oxide release could be minimized when they are used for biofuel production	The higher capital costs and the rather intensive care required by microalgal cultivation facility compared to a conventional agricultural farm would impede the commercial application of the biofuels from microalgae
4. *Strong adaptation to environment*	
Microalgae have strong adaptation to various environments without competing with fertile soils for agriculture	
5. *Growth in aqueous media, less water required*	
Microalgae do not need arable land to grow, they grow in aqueous media, therefore may not incur land-use change, minimizing the associated environmental impacts, and their cultivation consumes less water than terrestrial crops, thus reducing the load on freshwater sources	
6. *High carbohydrate content*	
Microalgae have high carbohydrate content, which is helpful in enhancing the hydrogen production efficiency	
7. *Simple structure*	
Microalgae are lack of hemicellulose and lignin, thus, the required pretreatments can be milder	
8. *Easy cultivation*	
Microalgae are unicellular or simple-multicellular microorganisms, which are adaptive to various environment conditions, and can be cultivated in fresh water, seawater and wastewater. The biochemical composition of the algal biomass can be modulated by varying growth conditions. The nutrients for microalgae cultivation (especially nitrogen and phosphorus) can be obtained from wastewater. Therefore, apart from providing growth medium, there is dual potential for treatment of wastewater	

One of the major disadvantages of microalgae for biohydrogen production is the low biomass concentration in the microalgal culture due to the limit of light penetration, which in combination with the small size of algal cells makes the harvest of algal biomass relatively costly. The large water content of harvested algal biomass also means its drying would be an energy-consuming process. The higher capital costs and the rather intensive care required by a microalgal farming facility compared to a conventional agricultural farm is another factor that impedes the commercial implementation of the biofuels from microalgae strategy.

Nevertheless, these problems are expected to be overcome or minimized by technology development. Given the vast potential of microalgae as the most efficient primary producers of biomass, there is little doubt that they will eventually become one of the most important alternative energy sources.

## Pretreatment of microalgal biomass

Since the hydrolytic enzymatic activity of hydrogen-producing bacteria is usually low, in order to enhance the biohydrogen production efficiency of fermentation process, the pretreatment step is often required for the hydrolysis of algal biomass to release the organic substances from the algal cells and make them readily biodegraded. A variety of pretreatment technologies that are researched and developed for treating other waste materials (e.g., animal waste and municipal sewage sludge) can be used to pretreat microalgal biomass for biohydrogen production.

Pretreatment methods can be divided into four categories: physical (mechanical, heat and ultrasonic treatment), chemical (acid, base and ozone), biological (enzymatic and microbiological treatment) and a combination of different treatments.

The most commonly used for pretreatment of microalgae to enhance carbohydrates hydrolysis include milling, ultrasonic, microwave, steam explosion, chemical oxidation and enzymatic hydrolysis.

In fact, the objective of all these pretreatment methods is the disruption of the cell wall to release the organic substances from the cells. Therefore they are applicable to biohydrogen production.

## Hydrogen production from microalgae

Microalgae have been used as feedstock for producing biodiesel, bioethanol and biogas. Various microbial species have been used as feedstock for biohydrogen production, among which *Chlorella* sp., *Scenedesmus* sp. and *Saccharina* sp. have been extensively studied. To enhance the hydrogen production efficiency, different pretreatment methods were explored.

### Hydrogen production from un-pretreated microalgae

Table 3 summarizes the hydrogen production from microalgae without pretreatment. It can be seen that *Chlorella vulgaris* is the most widely used as substrate for hydrogen production without treatment. Hydrogen yield obtained ranges from 0.37 to 19 mL H_2_/g VS, and highest hydrogen yield was achieved from *C. vulgaris* [15], followed by the lipid extracted *Scenedesmus* sp. [16].

**Table 3:** Hydrogen production from microalgae without pretreatment

Substrate	Substrate concentration (g/L TS)	Inoculum	Operational conditions	Hydrogen yield (mL H_2_/g VS)	Comments	References
*Chlorella vulgaris*	5	Anaerobic sludge	pH = 7.0, 37 °C; batch	10.8	Due to the activities of satellite bacteria associated with algal cultures, hydrogen can be produced with and without inocula. Addition of BESA inhibited both hydrogen production and methane production	[17]
*Chlorella vulgaris*	5–30	Anaerobic sludge	pH = 7.5, 60 °C; batch	1.75–19	Combination of hydrogen production from microalgae and methane production from hydrogen fermentation residues was investigated. Effects of different enzymatic pretreatment on hydrogen and methane yield were examined	[15]
*Chlorella vulgaris*	3–117	Anaerobic sludge	pH = 4.2–9.8, 35 °C; batch	14.6–31.2^b^	Hydrogen production from microalgae biomass via dark fermentation was optimized by response surface methodology (CCD). The optimal condition was found at 76 g TS/L and initial pH of 7.4	[18]
*Chlorella* sp.	4–40	Anaerobic sludge	pH = 6.5, 35 °C; batch	0.37–7.13	Influences of inoculum–substrate ratio, VFAs and NADH on anaerobic hydrogen production from *Chlorella* sp. were examined. Results showed that inoculum–substrate ratio and NADH had a negative correlation with hydrogen production and increase of VFA formation was accompanied with increased hydrogen production. 3D EEM fluorescence spectrometry was used to determine NADH	[19]
*Nannochloropsis* sp. NANNO-2	2.5–10	*Enterobacter aerogenes* ATCC 13048	30 °C; batch	26.4–60.6^b^	Hydrogen was produced from *Nannochloropsis* sp. biomass before or after lipid extraction. Higher hydrogen yield was obtained from lipid extracted microalgae biomass	[20]
*Nannochloropsis oceanica*	50	Anaerobic sludge	pH = 6.0, 35 °C; batch	2	The flue gas-cultivated microalgae biomass (*N*. *oceanica*) is efficiently used as feedstock to cogenerate hydrogen and methane through a novel three-stage method comprising dark fermentation, photo-fermentation and methanogenesis	[21]
*Scenedesmus* sp. (lipid extracted)	18^a^	Anaerobic sludge	pH = 6.3, 37 °C; batch	16.99	Different treatment methods on hydrogen production from microalgae biomass were examined. Include base, heat and combination of base and heat treatment. Treatment methods except base treatment all led to a significant increase in hydrogen production from microalgae biomass	[16]
*Scenedesmus* sp. (lipid extracted)	4.5–45^a^	Anaerobic sludge	pH = 5.0–7.0, 37 °C; batch	0.42–40.27	Effects of inoculum treatment, inoculum concentration, initial pH and substrate concentration on hydrogen production were investigated. Optimum condition was determined to be initial pH 6.0–6.5, heat treated inoculum concentration of 2.35 g VSS/L and the microalgae biomass concentration of 36 g VS/L	[22]
*Chlamydomonas reinhardtii*	50	*Clostridium butyricum* NCBI 9576	pH = 6.0, 37 °C; batch	16.6^b^	Anaerobic hydrogen production from *Chlamydomonas reinhardtii* biomass was followed by photo fermentation, increased hydrogen yield from 2.58 mol H_2_/mol starch–glucose to 8.30 mol H_2_/mol starch–glucose equivalent algal biomass	[23]
*Dunaliella tertiolecta*	5	Anaerobic sludge	pH = 7.0, 37 °C; batch	12.6	The high salinity of the *D. tertiolecta* slurry was prohibitive to methanogens, result in low methane production and high hydrogen yield	[17]

^a^g/L VS

^b^mL H_2_/g TS

### Hydrogen production from physically and chemically pretreated microalgae

The physical and chemical pretreatments, including mechanical, heat, ultrasonic, acid, base and ozonation, have been widely applied to disrupt and disintegrate the cell wall of microalgal biomass for enhancing the subsequent biological conversion process. For example, Ortigueira et al. [24] investigated fermentative hydrogen production using dry ground *Scenedesmus obliquus* biomass as feedstock. Usually, the use of microalgae biomass as a fermentable feedstock is determined by the recovery of the intracellular sugars and those that constitute the cell walls. Thermal pretreatment normally involves some additional pretreatment. For instance, when increasing temperature by autoclaving or microwaving, side pretreatments such as pressure build-up or electromagnetic radiation, respectively, will also have an effect on pretreated biomass. Chemical pretreatment of different types of wastes was shown to improve hydrogen production.

Table 4 summarizes the hydrogen production from microalgae pretreated by physical and chemical methods. It can be seen that hydrogen yield obtained from the physically and chemically treated microalgae were obviously higher than microalgae without treatment, indicating that both physical and chemical treatment can help to disrupt the microalgal cell. Relatively higher hydrogen yield was obtained from the heat treated microalgae (94.3–338 mL H_2_/g VS) and highest hydrogen yield was obtained from the heat treated *Chlorella sorokiniana* [25].

**Table 4:** Hydrogen production from microalgae pretreated by physical and chemical methods

Treatment methods	Substrate	Substrate concentration (g/L TS)	Inoculum	Operational conditions	Hydrogen yield (mL H_2_/g VS)	Comments	References
Milling	*Scenedesmus obliquus*	10–50	*Clostridium butyricum* DSM 10702	pH = 7.0, 37 °C; batch	28.1–35.0	Pure culture showed better hydrogen production than mixed culture	[24]
Milling	*Scenedesmus obliquus*	10–50	Anaerobic sludge	pH = 7.0, 37 °C; batch	5.4–34.8	Hydrogen production by mixed culture showed lower H_2_/CO_2_ ratio than pure culture	[24]
Milling	*Scenedesmus obliquus*	10–50	Anaerobic sludge	pH = 7.0, 58 °C; batch	0.7–15.3	Higher hydrogen production was achieved at higher temperature	[24]
Milling	*Scenedesmus obliquus*	10–50	Anaerobic sludge + *Clostridium butyricum* DSM 10702	pH = 7.0, 58 °C; batch	32.7–48.9	Co-culture of microorganisms achieved the highest hydrogen yield	[24]
Heat: 100 °C, 8 h	*Scenedesmus* sp. (lipid extracted)	18^a^	Anaerobic sludge	pH = 6.3, 37 °C; batch	35.38	Hydrogen production from microalgae biomass was increased by over 2 times after heat treatment at 100 °C for 8 h	[16]
Heat: 121 °C, 15 min	*Scenedesmus obliquus*	2.5–50	*Enterobacter aerogenes* ATCC 13048	pH = 6.8, 30 °C; batch	10.8–56.5	With the increase of substrate concentration, hydrogen yield decreased while cumulative hydrogen production and hydrogen production rate increased. Better hydrogen production was obtained from wet biomass than dried microalgae	[26]
Heat: 121 °C, 15 min	*Scenedesmus obliquus*	2.5–50	*Clostridium butyricum* DSM 10702	pH = 6.8, 37 °C; batch	94.3–113.1	Hydrogen yield, cumulative hydrogen production and hydrogen production rate increased with the increase of substrate concentration. Better hydrogen production was obtained from wet biomass than dried microalgae	[26]
Heat: 121 °C, 20 min	*Chlorella sorokiniana*	14	Anaerobic sludge	pH = 6.5, 60 °C; batch	338	Different treatment methods on hydrogen production from microalgae biomass were examined. XRD and SEM were used to examine the rupture effect on cells by different treatment methods	[26]
Heat: 121 °C, 4 h	*Scenedesmus* sp. (lipid extracted)	18^a^	Anaerobic sludge	pH = 6.3, 37 °C; batch	35.58	Increasing treating temperature from 100 to 121 °C can achieve similar hydrogen production but shorter treating time was needed	[16]
Base: NaOH 8 g/L, 24 h	*Scenedesmus* sp. (lipid extracted)	18^a^	Anaerobic sludge	pH = 6.3, 37 °C; batch	16.89	Base treatment alone showed little effect on hydrogen production from microalgae biomass	[16]
Chemical: H_2_O_2_ 2%, 12 h	*Chlorella sorokiniana*	14	Anaerobic sludge	pH = 6.5, 60 °C; batch	63	H_2_O_2_ showed better effect in treating microalgae biomass than sonication, but not as effective as other methods like heat and heat-acid treatment	[25]
Sonication: 130 W, 10 min	*Chlorella sorokiniana*	14	Anaerobic sludge	pH = 6.5, 60 °C; batch	52	Sonication showed little effect on cell disruption, and hydrogen production from sonication treated microalgae was not obviously increased	[25]

^a^g/L VS

### Hydrogen production from biologically pretreated microalgae

The biological approaches use microbes and enzymes to disrupt biomass and release intracellular materials, which enhances the biohydrogen production rate. Depending on cell wall composition, enzymes election is crucial. Additionally, pH, temperatures, and the microalgae/enzyme ratio are important parameters to control during enzymatic treatment. The electrostatic bind enzyme-microalgae are affected by acid or alkali conditions. Under inappropriate pH conditions, enzymes can even be inactivated by denaturing. Similarly, higher temperature results in increasing interactions enzyme-microalgae until a certain level at which denaturalization may happen. Finally, the enzyme/microalgae ratio influences the enzyme activity efficiency. High loading of microalgae may result in high viscosity due to the release of insoluble matter which in turn can hinder enzymatic activity.

Composition of microalgae cell walls include cellulose, mucopolysaccharide and peptidoglycan, etc., therefore research on microalgal biomass focus on the application of macerozyme. Cellulases were proven to be suitable for disruption of *C. sorokiniana* cell wall, and lysozyme was found to be able to dissolve Cyanobacteria cell wall. Cell wall lysis was supported by microscopic observation.

Table 5 summarizes the hydrogen production from microalgae pretreated by biological method. It can be seen that hydrogen yield varies greatly in the range of 11–135 mL H_2_/g VS. Higher hydrogen yield was obtained by enzyme treated microalgae than microbial consortium treated microalgae, and a combination of different enzymes can significant enhance the hydrogen yield [15].

**Table 5:** Hydrogen production from microalgae pretreated by biological methods

Treatment methods	Substrate	Substrate concentration (g/L TS)	Inoculum	Operational conditions	Hydrogen yield (mL H_2_/g VS)	Comments	References
Biological: Onozuka R-10 enzyme	*Chlorella vulgaris*	10	Anaerobic sludge	pH = 7.5, 60 °C; batch	39	Onozuka R-10 enzyme treatment increased hydrogen production from *Chlorella vulgaris* biomass from 19 to 39 mL/g VS	[15]
Biological: macerozyme R-10 enzyme	*Chlorella vulgaris*	10	Anaerobic sludge	pH = 7.5, 60 °C; batch	62	Macerozyme R-10 enzyme showed better effect on hydrogen production from *Chlorella vulgaris* biomass than Onozuka R-10 enzyme	[15]
Biological: Onozuka R-10 enzyme + macerozyme R-10 enzyme	*Chlorella vulgaris*	10	Anaerobic sludge	pH = 7.5, 60 °C; batch	135	Combination of Onozuka R-10 enzyme and macerozyme R-10 enzyme treatment resulted in significant increase in hydrogen yield from *Chlorella vulgaris* biomass than single enzyme treatment	[15]
Biological: microbial consortium TC60, 60 °C, 10 days	*Chlorella vulgaris*	0.14^a^	TC60 from compost	pH = 7.0, 60 °C; batch	11	*Chlorella* biomass showed recalcitrance to anaerobic digestion by TC60, and hydrogen was produced by satellite heterotrophs from *C. vulgaris*	[27]
Microbial consortium TC60, 60 °C, 10 days	*Dunaliella tertiolecta*	0.094^a^	TC60 from compost	pH = 7.0, 60 °C; batch	13	Hydrogen yields increased at least 10% after biological treatment process. Digestion of *Dunaliella tertiolecta* provided additional nutrients for cellulolytic activity	[27]

### Hydrogen production from microalgae pretreated by combined methods

To disintegrate biomass more efficiently and to take advantage of various pretreatment methods, the combination of different pretreatment methods has been used. Most combined pretreatment methods comprise a physical treatment method and a chemical treatment method.

Combined heat and acid pretreatment is the most commonly used method. Besides acid pretreatment, heat pretreatment has also been combined with other methods such as base pretreatment, enzymatic treatment and oxidizing agent addition. Other combination of pretreatment methods has been also applied, such as combining ozone with ultrasonication and enzyme hydrolysis, respectively; combining microwave with base and acid pretreatment, respectively; combining ionizing radiation and base pretreatment. All of them achieved enhanced hydrogen production from pretreated biomass wastes. In some cases, combinations of three or more pretreatment methods were also used, such as acid-heat-enzyme pretreatment [28, 29], acid-microwave-enzyme pretreatment [30], base-heat-enzyme pretreatment [31] and so on.

Table 6 summarized the hydrogen production from microalgae pretreated by the combined methods. It can be seen that the hydrogen yield varies in the range of 33.56–958 mL H_2_/g VS. The combination of acid and heat showed the highest potential in enhancing the hydrogen production from microalgae, and *Chlorella* sp. are more preferable in achieving higher hydrogen yield.

**Table 6:** Hydrogen production from microalgae pretreated by combined methods

Treatment methods	Substrate	Substrate concentration (g/L TS)	Inoculum	Operational conditions	Hydrogen yield (mL H_2_/g VS)	Comments	References
Acid: HCl 2.0%, 12 h; Heat: 121 °C, 20 min	*Chlorella sorokiniana*	10	*Enterobacter cloacae* IIT-BT 08	pH = 7.0, 37 °C; batch	201.6^c^	Algal biomass of *C. sorokiniana* was produced by CO_2_ sequestration in continuous mode, and then used as substrate for anaerobic hydrogen production. Substrate concentration was optimized to enhance the hydrogen yield from *C. sorokiniana*	[32]
Acid-heat: HCl 5%, 121 °C, 20 min	*Chlorella sorokiniana*	14	Anaerobic sludge	pH = 6.5, 60 °C; batch	760	Better hydrogen production was achieved from microalgae biomass treated by combined treatment than single treatment method including autoclave, sonication and H_2_O_2_ treatment	[25]
Acid-heat: HCl 20%, 121 °C, 20 min	*Chlorella sorokiniana*	14	Anaerobic sludge	pH = 6.5, 60 °C; batch	958	Hydrogen yield was increased from 760 to 958 mL/g VS when HCl concentration was increased from 5 to 20%	[25]
Acid-heat: H_2_SO_4_ 0.1 mM, 108 °C, 30 min	*Chlorella vulgaris*	20	*Clostridium acetobutylicum* B-1787	pH = 6.8, 37 °C; batch	2.24^d^	Immobilized *Clostridium acetobutylicum* cells were used for hydrogen production from various microalgae species	[33]
Acid-heat: H_2_SO_4_ 0.1 mM, 108 °C, 30 min	*Nannochloropsis* sp. rsemsu-N-1	20	*Clostridium acetobutylicum* B-1787	pH = 6.8, 37 °C; batch	0.90–9.52^d^	Different microalgae species were used as substrate, and highest hydrogen yield was obtained from wet *Nannochloropsis* sp. biomass	[33]
Acid-heat: H_2_SO_4_ 0.1 mM, 108 °C, 30 min	*Arthrospira platensis*	20	*Clostridium acetobutylicum* B-1787	pH = 6.8, 37 °C; batch	2.24–8.06^d^	Heating temperature range of 100–121 °C, with and without acid addition were applied in treating microalgae biomass, most efficient treatment condition was determined to be 108 °C, 30 min with 0.1 mmol/L H_2_SO_4_	[33]
Acid-heat: H_2_SO_4_ 0.1 mM, 108 °C, 30 min	*Dunaliella tertiolecta*	20	*Clostridium acetobutylicum* B-1787	pH = 6.8, 37 °C; batch	0.22–1.46^d^	Immobilized *Clostridium acetobutylicum* cells were used for hydrogen production from various microalgae species	[33]
Acid-heat: H_2_SO_4_ 0.5 mol/L, 100 °C, 30 min	*Scenedesmus obliquus*	–	*Clostridium butyricum*	pH = 7.0, 37 °C; batch	2.9^e^	Potential of H_2_ production from microalgae biomass and the respective energy consumption and CO_2_ emissions in the bioconversion process were evaluated. Energy consumption of 7270 MJ/MJH_2_ and 670 kg CO2/MJH_2_ were achieved, 98% of which owed to microalgae culture process due to the use of artificial lighting	[34]
Acid-heat: H_2_SO_4_ 0.5%, 121 °C, 60 min	*Spirulina platensis*	10	*Bacillus firmus* NMBL-03	pH = 6.5, 38 °C; batch	0.38^e^	A wide variety of substrates (glucose, xylose, arabinose, lactose, sucrose, and starch) and carbohydrate rich waste products (bagasse hydrolysate, molasses, potato peel and *cyanobacterial* mass) were used for dark fermentative hydrogen production. Abundant VFA were present in spent medium of hydrogen production from *cyanobacterial* mass, which can be further used as substrate for photo fermentative hydrogen production	[35]
Acid-heat: H_2_SO_4_ 1%, 135 °C, 15 min	*Chlorella pyrenoidosa*	20	*Clostridium butyricum*	pH = 6.0, 35 °C; batch	81.2	Heat and acid treated *Chlorella pyrenoidosa* biomass was used as substrate for hydrogen production. Energy was further removed through following photo hydrogen production and methane fermentation	[36]
Acid-heat: H_2_SO_4_ 1%, 135 °C, 15 min	*Chlorella pyrenoidosa*	10 (additional cassava starch 10 g/L)	*Clostridium butyricum*	pH = 6.0, 35 °C; batch	276.2	Hydrogen production from microalgae biomass was significantly increased from 81.2 to 276.2 mL/g VS by the addition of cassava starch to get an optimum C/N ratio	[36]
Acid-heat: H_2_SO_4_ 3%, 121 °C, 60 min	Lipid extracted algae cake (collected from a lake)	5^b^	Anaerobic sludge	pH = 6.0, 29 °C; batch	122^d^	Comparison of hydrogen production from algae untreated, liquid fraction of treated algae, solid fraction of treated algae and treated algae mixture was examined. Best hydrogen and VFA generation was achieved from liquid fraction of treated algae	[37]
Acid-microwave: H_2_SO_4_ 0–2.0%, 80–180 °C, 5–25 min	*Nannochloropsis oceanica*	50	Anaerobic sludge	pH = 6.0, 35 °C; batch	39	Hydrogen production from microalgae biomass was significantly increased by combined acid and microwave treatment	[21]
Base-heat: NaOH, 8 g/L, 100 °C, 8 h	*Scenedesmus* (lipid extracted)	18^a^	Anaerobic sludge	pH = 6.3, 37 °C; batch	45.54	For the combined treatment, lower temperature and longer treating time was preferred than higher temperature and shorter time	[15]
Base-heat: NaOH, 8 g/L, 121 °C, 4 h	*Scenedesmus* (lipid extracted)	18^a^	Anaerobic sludge	pH = 6.3, 37 °C; batch	37.42	Better hydrogen production was achieved from microalgae biomass treated by combined treatment than single treatment method	[16]
Acid-heat: pH 1.4, 140 °C, 15 min; biological: cellulase 0.05 g/g TVS, 48 h; glucoamylase 0.05 g/g VS, 24 h	Mixed algae (collected from algae bloom in Taihu Lake)	25	Anaerobic sludge	pH = 6.0, 35 °C; batch	33.56–43.84	Steam with acid treatment showed better reducing sugar release than steam with alkaline treatment. The energy conversion efficiency was significantly increased through 3-stage process: dark-fermentation, photo-fermentation, and methanogenesis	[30]
Acid-microwave: pH 1.4, 140 °C, 15 min; biological: cellulase 0.05 g/g TVS, 48 h; glucoamylase 0.05 g/g TVS, 24 h	Mixed algae (collected from algae bloom in Taihu Lake)	25	Anaerobic sludge	pH = 6.0, 35 °C; batch	42.4–47.07	Microwave with diluted acid treatment degraded algal cells into smaller fragments (< 5 mm), and resulted in higher saccharification efficiency of microalgae	[30]
Acid-microwave: H_2_SO_4_ 0.2 mL, 140 °C, 15 min; biological: glucoamylase 0.2%	*Arthrospira platensis*	10–40	Anaerobic sludge	pH = 6.5, 35 °C; batch	86.5–96.6^d^	Hydrogen yield was significantly enhanced from 96.6 to 337.0 mL H_2_/g DW using a combination of dark- and photo-fermentation. Removal of harmful byproducts from hydrolysis pretreatment and dark fermentation can further enhance the overall hydrogen yield	[38]

^a^g/L VS

^b^g/L COD

^c^mL H_2_/g COD

^d^mL H_2_/g TS

^e^mol H_2_/mol sugar

## Concluding remarks and perspectives

Microalgae are capable of producing high levels of carbohydrates such as starch or cellulose as reserve materials, which are ideal feedstocks for hydrogen production. Microalgae can potentially be employed for the production of biohydrogen in an economically affective and environmentally sustainable manner. The production of biohydrogen from microalgae can be integrated with flue gas (CO_2_) mitigation, wastewater treatment, and the production of high-value chemicals. There is increasing interest in using microalgae as the renewable feedstock for the production of biohydrogen. In comparison with terrestrial biofuel feedstocks, microalgae can convert solar energy into fuels with higher photosynthetic efficiency, can synthesize and accumulate large quantities of carbohydrate biomass, and can thrive in seawater system.

Studies have shown that fermentative hydrogen production from microalgae shows great potential in sustainable energy generation. Hydrogen production can be modified through disrupting the microalgal cells by some pretreatment methods, and a proper combination of different treatment methods can achieve a synergistic effect and thus significantly enhance the hydrogen yield.

However, there still remain some obstacles hindering the wide application of hydrogen production from microalgae, and several technological and economic issues must be addressed to achieve success on a commercial scale. Studies have shown great variance in the hydrogen yield, some of the hydrogen yields are high, like 958 and 760 mL H_2_/g VS obtained from acid-heat treated *C. sorokiniana* while some are far from industrial application. Thus, further studies are needed to enhance the cost effectiveness of the biohydrogen from microalgae, like the improvement in microalgal cultivation and downstream processing (e.g., harvesting, concentrating and drying), optimization of nutritional structure of microalgae for hydrogen production through adding protein-rich or mineral nutrient-rich wastes, operational conditions optimization including inoculum, initial pH, temperature as well as reactor structure, etc.
